# A meta-analysis of soil microbial biomass responses to forest disturbances

**DOI:** 10.3389/fmicb.2013.00163

**Published:** 2013-06-20

**Authors:** Sandra R. Holden, Kathleen K. Treseder

**Affiliations:** Department of Ecology and Evolutionary Biology, University of CaliforniaIrvine, CA, USA

**Keywords:** disturbance, fire, forest, harvest, insect, soil microbial biomass, pathogen, storm

## Abstract

Climate warming is likely to increase the frequency and severity of forest disturbances, with uncertain consequences for soil microbial communities and their contribution to ecosystem C dynamics. To address this uncertainty, we conducted a meta-analysis of 139 published soil microbial responses to forest disturbances. These disturbances included abiotic (fire, harvesting, storm) and biotic (insect, pathogen) disturbances. We hypothesized that soil microbial biomass would decline following forest disturbances, but that abiotic disturbances would elicit greater reductions in microbial biomass than biotic disturbances. In support of this hypothesis, across all published studies, disturbances reduced soil microbial biomass by an average of 29.4%. However, microbial responses differed between abiotic and biotic disturbances. Microbial responses were significantly negative following fires, harvest, and storms (48.7, 19.1, and 41.7% reductions in microbial biomass, respectively). In contrast, changes in soil microbial biomass following insect infestation and pathogen-induced tree mortality were non-significant, although biotic disturbances were poorly represented in the literature. When measured separately, fungal and bacterial responses to disturbances mirrored the response of the microbial community as a whole. Changes in microbial abundance following disturbance were significantly positively correlated with changes in microbial respiration. We propose that the differential effect of abiotic and biotic disturbances on microbial biomass may be attributable to differences in soil disruption and organic C removal from forests among disturbance types. Altogether, these results suggest that abiotic forest disturbances may significantly decrease soil microbial abundance, with corresponding consequences for microbial respiration. Further studies are needed on the effect of biotic disturbances on forest soil microbial communities and soil C dynamics.

## Introduction

Forest ecosystems are a critical component of the global carbon (C) cycle. Boreal, temperate, and tropical forests cover ~30% of the global land surface and store ~1600 Pg C, accounting for up to 45% of global terrestrial carbon (Bonan, 2008). Forests are subject to frequent stand disturbances that can alter the amount of C stored in forests. For example, forest fires burn an average of ~40,000 km^2^ in North American forests (Giglio et al., 2006), and ~2,000 km^2^ in European forests each year (Schelhaas et al., 2003). Likewise, ~50,000 km^2^ of North American forests are harvested annually (Birdsey et al., 2006). Other common forest disturbances include storms, insect outbreaks, and pathogen infection of trees (Goetz et al., 2012). These disturbances can be grouped into abiotic (fire, harvesting, storm) and biotic (insect, pathogen) disturbances. Although already common, some forest disturbances may occur more frequently and severely as a result of climate warming. For example, modeling studies predict that the burned area in Alaskan and Canadian boreal forests will increase 3.5–5.5 times by the end of the century (Balshi et al., 2009). Higher temperatures may also provide more favorable conditions for insects and pathogens, and make forests more susceptible to infestation (Dale et al., 2001). Although, insect outbreaks are not always directly related to climatic conditions (Kardol et al., 2010). Given the large amount of C stored in forests, it is important to understand how disturbances alter ecosystem C dynamics.

Soil microbial respiration of CO_2_, produced as a result of organic matter decomposition in soil, comprises a large flux of C from forest ecosystems to the atmosphere. Classic ecosystem theory predicts that the total amount of CO_2_ released by soil microbes increases following forest disturbances (Odum, 1969; Chapin et al., 2002), owing to post-disturbance increases in soil temperature and C availability. Direct *in situ* measurements of microbial respiration following disturbances are scarce (but see Czimczik et al., 2006). Indirect evidence for increased microbial respiration following disturbances is derived primarily from measurements of soil C stocks (Covington, 1981) and from measurements of total soil respiration (Richter et al., 2000). However, despite the central role of microbes in decomposition and C release from soils, the response of soil microbial biomass and community composition to forest disturbances is not accounted for in this classic ecosystem theory.

Abiotic and biotic disturbances change a variety of soil properties in forests, which may in turn alter soil microbial biomass and respiration. For example, abiotic disturbances usually kill (fire, storm) or remove (harvest) aboveground vegetation. Post-disturbance reductions in aboveground vegetation decrease plant litter inputs and root exudation into soil and thus can result in long-term declines in soil C (Johnson and Curtis, 2001; Wang et al., 2012; Zhou et al., 2013) and total soil nitrogen (Wan et al., 2001). In addition, soil temperatures often increase following abiotic disturbances (Treseder et al., 2004), and this may augment microbial respiration. However, microbes living in post-disturbance soils may also experience greater moisture stress, as higher soil temperatures following abiotic disturbance can lead to soil drying. Biotic disturbances may differ from abiotic disturbances in their effect on soil properties because they less frequently kill aboveground vegetation. Tree defoliation caused by biotic disturbances can result in an influx of dead plant litter into soils (Hicke et al., 2012). Insect biomass and frass deposition following insect defoliation can also increase soil nutrient availability (Lovett et al., 2002). Increases in labile C and nutrient availability following biotic disturbances may stimulate soil microbial growth and respiration. On the other hand, biotic disturbances that kill aboveground vegetation might cause soil C availability to decline. The net effect of these altered soil conditions on soil microbial communities is poorly understood.

Soil microbial responses to forest disturbances are likely to differ as a function of the time since disturbance. Disturbance effects on soil microbial communities may only persist until aboveground vegetation re-grows, as the recovery of aboveground vegetation may reverse changes in soil properties caused by disturbance (Hart et al., 2005). Soil nutrient availability may quickly return to pre-disturbance levels if soil microbes and plants can readily assimilate the pulse of available nutrients. Furthermore, soil microbial communities may have the capacity to quickly recover from disturbances if nearby undisturbed forests or mineral soils serve as a source of microbial inoculum (Grogan et al., 2000; Barker et al., 2013). However, we currently have a limited understanding of changes in soil microbial biomass during forest recovery from a variety of disturbance types.

In a previous meta-analysis we summarized soil microbial biomass responses to fire (Dooley and Treseder, 2012). This work demonstrated that fires reduce soil microbial biomass in forest ecosystems. However, our previous work did not examine other types of forest disturbances besides fire. It is important to consider microbial responses to a variety of disturbances because of their prevalence in forests worldwide and the likelihood that disturbances may occur more frequently as a result of climate warming. Determining the relative impact of different disturbance types will allow us to better predict how climate-linked increases in disturbance frequency will affect soil microbial communities and soil C dynamics. Many studies have documented soil microbial responses to forest disturbances, but the results among these studies are inconsistent. Some studies find increases in microbial abundance following disturbances (Holmes and Zak, 1999; Bogorodskaya et al., 2009), while others report negative microbial responses to disturbance (Arunachalam et al., 1996; Bárcenas-Moreno et al., 2011) and we lack a quantitative synthesis across disturbance types. Here, we build on our previous work by asking how does soil microbial biomass and respiration respond to disturbance events in forests and how does this response differ across disturbance types? We also highlight forest disturbance types that require further study. We hypothesized that forest disturbances would reduce soil microbial biomass. Second, we expected that abiotic disturbances would lead to greater reductions in microbial biomass than biotic disturbances. Third, we predicted that post-disturbance changes in microbial biomass would diminish over time as forests recover from disturbance. Fourth, we expected that changes in soil microbial biomass would be associated with changes in microbial respiration. We tested these hypotheses separately for studies that measured total soil microbial biomass, and for studies that measured fungal and bacterial abundances separately since these major classes of microbes may have different responses to disturbance. Given previous work suggesting that fungi may be more sensitive to fires than bacteria (Pietikäinen and Fritze, 1995; Dooley and Treseder, 2012), we expected that fungi would have larger responses to disturbance than bacteria.

## Materials and methods

### Literature survey and criteria for inclusion

We searched the published literature for studies that reported microbial abundance measurements in disturbed and undisturbed forest soils. Searches were conducted using the ISI Web of Science database and Google Scholar. We performed our literature searches separately by each type of forest disturbance. Key words for each disturbance type included: burn, forest fire, prescribed fire, wildfire (fire); harvest, logging (forest harvest); insect, insect defoliation, insect outbreak (insect outbreaks); pathogen (pathogen-caused tree mortality); and storm, windthrow (storms). To narrow our search results to studies that focused on soil microbes, we also used the search terms microb*, bacteri*, and fung* in combination with the key words listed above for each disturbance type. Published studies were collected for analysis until 15 January 2013.

Meta-analyses were preformed on a subset of studies that met our search criteria (Table A1) following Dooley and Treseder (2012). Importantly, we only included multiple data sets from a single study if the data sets could reasonably be considered independent (e.g., different geographic locations, dominant vegetation).

### Data acquisition

For each study, we recorded the mean, standard deviation (SD), and sample size (*n*) of microbial biomass, fungal abundance, or bacterial abundance in the disturbed area and the undisturbed control. In addition to changes in microbial abundances, we recorded the type of disturbance, the disturbance agent, the time elapsed since disturbance, and the biome in which the study took place. We included studies from boreal forests, temperature forests, tropical forests, and woodlands. Studies in woodlands were primarily from Mediterranean ecosystems and had decreased tree biomass and higher amounts of shrub biomass. We also recorded the method used for measuring microbial abundances in soil. When means and errors were presented in graph form, we digitized the data using PlotDigitizer 2.6.2 (http://plotdigitizer.sourceforge.net). If standard errors (SEs) were presented instead of SDs, they were converted using the formula: *SD* = *SE* (*n*^1/2^). Any unidentified errors bars in graphs were assumed to represent SEs. There were a total of two studies in which error bars were not identified (Chang et al., 1995; Pietikäinen and Fritze, 1995).

### Indices of microbial abundance

Authors employed a variety of techniques to measure microbial abundances in soil. Microbial biomass in soil was measured through chloroform fumigation and extraction (Brooks et al., 1985), substrate-induced respiration (Anderson and Domsch, 1978), total amounts of phospholipid fatty acids (PLFAs) in soil (Frostegard and Bååth, 1996), total amounts of ATP extracted from soil (Eiland, 1983), and microwave irradiation of soil (Islam and Weil, 1998). Fungal abundance in soil was most commonly determined using fungal specific PLFAs. Additional methods for characterizing fungal abundance included total amounts of ergosterol in soil (Djajakirana et al., 1996), microscopy, plating soil and counting colony formation, and quantitative PCR with universal fungal primers (Borneman and Hartin, 2000). Bacterial abundances were determined through bacteria specific PLFAs, dilution plating, and microscopy.

### Specific microbial groups

A subset of the studies generated from our literature search also reported changes in the abundance of specific groups of bacteria in response to disturbance. We found studies that reported the response of gram-negative bacteria, gram-positive bacteria, and actinomycetes to forest disturbances. The abundance of these bacterial groups was measured using PLFAs or dilution plating.

### Basal respiration

Where possible, we also recorded changes in soil basal respiration following disturbances. We defined basal respiration as the amount of CO_2_ produced during laboratory incubations of soil in the absence of carbon or nutrient additions.

### Statistics

Meta-analyses were used to determine the significance of microbial abundance responses to disturbance. For each study and group of microorganisms (microbes, fungi, bacteria, gram-negative, gram-positive, actinomycetes), the effect size was calculated at the natural log of the response ratio (“*R*”). *R* is calculated as the mean of the disturbed treatment divided by the mean of the control group. Thus, an *R* of 1 indicates that disturbance had no effect on microbial abundance. Variance within each study (ν_*lnR*_) is computed using the means, *n*, and SD of the control and disturbed groups (Hedges et al., 1999).

To determine if disturbances had a significant effect on microbial abundance, we employed a random effects models using MetaWin software (Rosenberg et al., 2000). Bias-corrected bootstrap 95% confidence intervals (CIs) were calculated for each mean *R*. If the 95% CIs of *R* do not overlap with 1, then responses were significant at *P* < 0.05. Random effects models allow for comparisons between groups in a framework that is similar to analysis of variance. We applied random effects meta-analyses to test for differences in *R* between abiotic and biotic disturbances and disturbance types (fire, harvest, storm, insect, pathogen). Within each disturbance type, we further tested for differences among disturbance agents (e.g., wildfire vs. prescribed fire), biomes, and the method of measurement used to estimate microbial abundances. In addition, we used continuous randomized effects meta-analyses to test for relationships between *R* and the time since disturbance. Tests for the relationship between *R* and the time since disturbance were performed separately for each disturbance type and biome. Statistical results reported include: *R*, 95% CIs for *R*, and total heterogeneity in *R* among studies (*Q*_*T*_). For comparisons among groups, total heterogeneity (*Q*_*T*_) can be partitioned into the amount of heterogeneity explained by groups (*Q*_*M*_) and the amount of heterogeneity left unexplained (*Q*_*E*_). The significance of *Q*_*T*_ and *Q*_*M*_ is tested by comparison to the chi-squared distribution. A significant *Q*_*T*_ value means that the variance among studies is greater than expected due to sampling error. A significant *Q*_*M*_ values indicates that a significant portion of the total heterogeneity among studies can be explained by subdividing the studies into the group of interest (Rosenberg et al., 2000, 2004; Koricheva et al., 2013). We used a Pearson's correlation to analyze the relationship between the *R* of microbial biomass and the *R* of basal respiration for studies in which both were reported.

We employed a number of complementary approaches to test for the presence of publication bias in our data. We performed a Kendall's tau rank correlation test and a Spearman rank correlation test (Sokal and Rohlf, 1995) to test for the relationship between replicate number of each study and the standardized effect size. Such a relationship would be indicative of a publication bias in which larger effects of disturbance were more likely to be published than smaller effects. We visually inspected funnel plots of standard error or replicate number versus standardized effect size for the presence of asymmetry (Egger et al., 1997; Sterne and Egger, 2001). Funnel plot asymmetry was formally tested using Egger's regression (Sterne and Egger, 2005). Publication bias was assessed in all data for a given group of microorganisms (microbes, fungi, bacteria) and also for abiotic and biotic data sets within each group of microorganisms.

## Results

In this study we focused on five of the most prevalent disturbances in forest ecosystems. Specifically, we focused on three abiotic disturbances (fire, harvest, and storms) and two biotic disturbances (insect infestation and pathogen infection). Each disturbance type was further separated into its causative disturbance agent. Fires were grouped into wildfires, prescribed fires, or slash burns. Harvesting was grouped into clear cutting or partial harvesting (e.g., thinning, selective harvesting). Storms were subdivided into hurricanes, typhoons, and windthrow. We found studies reporting insect infestation by the gypsy moth, hemlock wooly adelgid, pine beetle, and pine lappet. Pathogen infection studies reported the effects of pine wilt disease and *Phellinus weirii* infection. Our literature search produced 88 observations of changes in soil microbial biomass following forest disturbances, collected from a total of 61 published papers. We found 35 reports of fungal abundance responses to disturbance from 24 published studies. Finally, we found 16 observations of changes in bacteria abundance following disturbance from 12 published papers.

### Total microbial biomass

**Figure 1:**
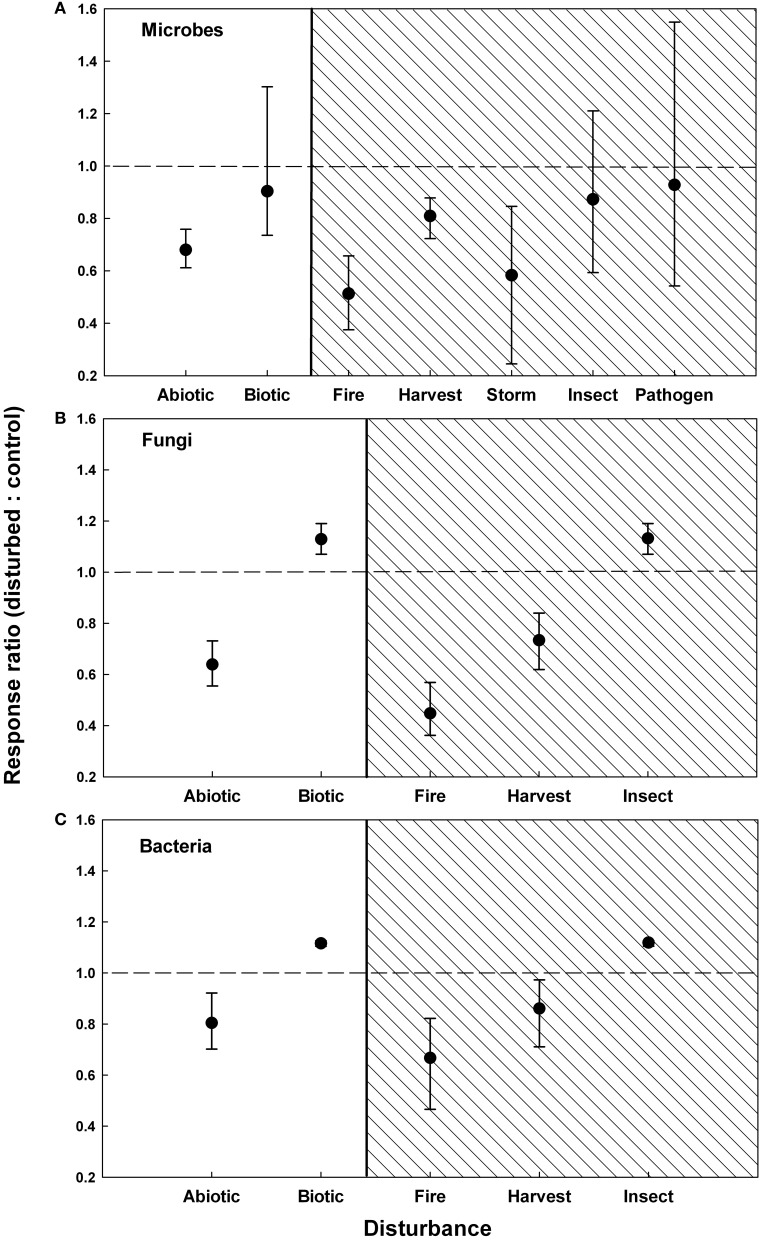
**Responses of microbial biomass (A), fungal abundance (B), and bacterial abundance (C) to forest disturbances**. Response ratios are grouped by abiotic and biotic disturbances (unshaded) and by disturbance type (shaded). Symbols are means ± 95% confidence intervals. A response ratio < 1 indicates that microbial abundances declined following disturbance, a response ratio > 1 indicates an increase in microbial biomass.

Soil microbial biomass significantly decreased following disturbances, by an average of 29.4% across all studies (Table 1). However, disturbance responses were not consistent across studies, as indicated by a significant *Q*_*T*_ value (*Q*_*T*_ = 110.95, *P* = 0.043). Microbial biomass responses to disturbance differed significantly between abiotic and biotic disturbances (*Q*_*M*_ = 14.68, *Q*_*E*_ = 99.45, *P* = 0.038, Figure 1A). Fires, harvesting, and storms resulted in significant reductions in microbial biomass (by 48.7, 19.1, and 41.7%, respectively). In contrast, changes in soil microbial biomass following insect attack and pathogen-induced mortality were non-significant (Figure 1A).

**Table 1:** **Results of statistical comparisons among and within groups**.

**Organism**	**Group**	**Sub-group**	***R***	**95% CI**	**Number of studies**	***Q*_*M*_**	***Q*_*E*_**	***P*-value groups^a^**
Microbes	All microbe studies^*^		0.71	0.63–0.80	88			
	Abiotic	All abiotic studies^*^	0.68	0.61–0.76	80			
	Fire	All fire studies^*^	0.51	0.38–0.66	28			
	Fire Type	Prescribed fire^*^	0.65	0.47–0.87	13	2.79	29.86	0.160
		Wildfire^*^	0.41	0.23–0.60	15			
	Biome	Boreal forest^*^	0.46	0.35–0.60	7	6.14	26.26	0.110
		Temperate forest^*^	0.35	0.19–0.57	11			
		Woodland/shrubland	0.79	0.53–1.09	10			
	Measurement	Chloroform fumigation^*^	0.46	0.31–0.64	21	3.44	27.17	0.303
		PLFA^*^	0.72	0.65–0.84	3			
		SIR^*^	1.17	1.06–1.29	2			
	Harvest	All harvest studies^*^	0.81	0.72–0.88	49			
	Harvest type	Clear cut^*^	0.78	0.67–0.86	34	1.23	42.01	0.315
		Partial harvest	0.89	0.78–1.02	13			
	Biome	Boreal forest^*^	0.87	0.81–0.94	20	1.76	46.37	0.434
		Temperate forest^*^	0.77	0.63–0.90	24			
		Tropical forest^*^	0.75	0.51–0.97	5			
	Measurement	Chloroform fumigation^*^	0.79	0.58–0.93	21	2.12	47.85	0.511
		PLFA^*^	0.90	0.81–0.98	11			
		SIR^*^	0.79	0.70–0.90	13			
	Storm	All storm studies^*^	0.58	0.25–0.85	3			
	Biotic	All biotic studies	0.90	0.74–1.30	8			
	Insect	All insect studies	0.87	0.59–1.21	6			
	Insect type	Gypsy moth^*^	1.46	1.42–1.51	2	28.23	2.51	0.102
		Pine beetle^*^	0.59	0.37–0.65	3			
	Biome	Boreal forest^*^	1.46	1.42–1.51	2	7.07	4.08	0.061
		Temperate forest^*^	0.68	0.44–0.92	4			
	Measurement	Chloroform fumigation^*^	0.68	0.44–0.92	4	7.07	4.08	0.061
		SIR^*^	1.46	1.42–1.51	2			
	Pathogen	All pathogen studies	0.93	0.54–1.55	2			
Fungi	All fungi studies^*^		0.66	0.57–0.76	35			
	Abiotic	All abiotic studies^*^	0.64	0.56–0.73	33			
	Fire	All fire studies^*^	0.45	0.36–0.57	13			
	Fire Type	Prescribed fire^*^	0.41	0.35–0.51	7	0.02	11.89	0.864
		Wildfire^*^	0.43	0.31–0.56	5			
	Biome	Boreal forest^*^	0.37	0.31–0.41	4	2.53	10.00	0.241
		Temperate forest^*^	0.55	0.35–0.78	5			
		Woodland/shrubland^*^	0.50	0.35–0.61	4			
	Measurement	Dilution plate count^*^	0.53	0.03–0.63	3	16.04	8.54	0.066
		Ergosterol^*^	0.36	0.30–0.42	2			
		Microscopy^*^	0.74	0.60–0.89	3			
		PLFA^*^	0.37	0.34–0.46	4			
	Harvest	All harvest studies^*^	0.73	0.62–0.84	20			
	Harvest type	Clear cut^*^	0.70	0.60–0.80	15	1.44	17.20	0.249
		Partial harvest	0.86	0.60–1.14	5			
	Biome	Boreal forest^*^	0.84	0.75–0.91	11	22.46	34.39	0.015
		Temperate forest^*^	0.71	0.52–0.95	7			
		Tropical forest^*^	0.45	0.45–0.45	2			
	Measurement	Dilution plate count	0.68	0.45–1.01	4	1.18	14.64	0.562
		Microscopy^*^	0.62	0.47–0.75	3			
		PLFA^*^	0.79	0.65–0.94	12			
	Biotic	All biotic studies^*^	1.13	1.07–1.19	2			
	Insect	All insect studies^*^	1.13	1.07–1.19	2			
Bacteria	All bacteria studies^*^		0.85	0.73–0.95	16			
	Abiotic	All abiotic studies^*^	0.81	0.70–0.92	14			
	Fire	All fire studies^*^	0.67	0.47–0.82	4			
	Harvest	All harvest studies^*^	0.86	0.71–0.97	10			
	Harvest type	Clear cut^*^	0.89	0.70–0.98	8	4.25	58.96	0.369
		Partial harvest	0.74	0.63–1.52	2			
	Biome	Temperate forest	0.99	0.96–1.01	7	132.14	18.96	0.020
		Tropical forest^*^	0.60	0.57–0.63	2			
	Measurement	Dilution plate count	0.74	0.57–1.00	3	15.69	32.89	0.278
		Microscopy	0.99	0.98–1.01	3			
		PLFA	0.88	0.70–1.52	3			
	Biotic	All biotic studies^*^	1.12	1.11–1.13	2			
	Insect	All insect studies^*^	1.12	1.11–1.13	2			

*PLFA, phospholipid fatty acid; SIR, substrate induced respiration*.

* *Significant effect of disturbance on group (P < 0.05)*.

a *Only groups represented by two or more studies were included in comparisons*.

Fire, harvest, and insect infestation had high enough replication in the literature to further test for differences among groups within each disturbance type. Within fires, microbial biomass response ratios were not significantly different among fire types, biomes, or measurement methods (Table 1). Fires in boreal and temperate forests significant reduced microbial biomass, but woodland fires had non-significant effects.

Following forest harvest, the response of microbial biomass was not significantly different between harvest types, biomes, or measurement methods (Table 1). Forest clear cutting elicited a significant negative response from soil microbes. In contrast, partial harvesting did not significantly alter soil microbial biomass. Within insect studies, infestation by pine beetles resulted in a significant reduction in soil microbial biomass, while defoliation by the gypsy moth significant increased soil microbial biomass (Table 1). Studies following storms (*n* = 3) and pathogen infection (*n* = 2) were scarce in the literature and thus we could not perform further comparisons within these disturbance types.

We performed tests for publication bias separately for each group of microorganisms (microbes, fungi, bacteria). Within each group, we also performed tests separately for abiotic and biotic studies. Across all microbe studies, we did not detect significant publication bias with any of the three tests used (Table A2). However, when abiotic and biotic disturbances were examined separately, Egger's regression test was significant for biotic disturbance studies (Table A2). This indicates a potential bias toward publishing significant results.

### Fungi

Across all studies, disturbances resulted in a 34.0% reduction in fungal abundance (Table 1). Abiotic and biotic disturbances had significantly different effects on fungal biomass (*Q*_*M*_ = 16.45, *Q*_*E*_ = 30.93, *P* = 0.008, Figure 1B). Fire and harvest resulted in 55.2 and 26.6% declines in soil fungi, respectively. Responses of fungi to insect infestation were significantly positive (Figure 1B). However, it is important to note that insect infestations were only represented by two observations in the literature.

Within fire studies, fungal responses were significantly negative, regardless of fire type, biome, or measurement method (Table 1). Within harvest studies, fungal responses were significantly different across biomes. Harvesting in tropical forests led to greater reductions in fungal biomass than harvesting in either boreal forests or temperate forests. Harvest responses did not differ by harvest type or measurement method. Similar to total soil microbial biomass, clear cutting significantly reduced fungal biomass, but partial harvesting had non-significant effects.

The Kendall's Tau and Spearman rank correlation tests for publication bias were significant for all fungal studies and for fungal studies of abiotic disturbances. However, Egger's regression test detected no significant publication bias for these same studies (Table A2). Our data set contained only two observations of changes in fungal abundance in response to biotic disturbances. Thus, we could not test for publication bias within biotic disturbances for fungi using correlation or regression methods.

### Bacteria

Bacterial abundance declined by an average of 15.3% in response to disturbances (Table 1). Bacterial responses to disturbance differed significantly between abiotic and biotic disturbances (*Q*_*M*_ = 29.53, *Q*_*E*_ = 66.45, *P* = 0.037, Figure 1C). Fire and harvest reduced bacteria by 33.3% and 13.9%, respectively. In contrast, bacteria increased following insect infestation (Figure 1C). Harvesting was the only disturbance type with sufficient replication to further test for differences within harvest studies. Bacteria harvesting responses were significantly different across biomes (Table 1). Harvesting in tropical forests significantly reduced bacterial biomass, but responses in temperate forests were non-significant. There were no significant differences in bacterial responses among harvest types and measurement methods. Clear-cutting significantly lowered soil bacterial abundance, but there was no significant effect of partial forest harvest.

A small subset of the studies included in this meta-analysis reported the response of specific groups of bacteria to disturbance (Table A1). Across all of these studies, we found that disturbances significantly reduced the abundance of gram-positive (*n* = 5, 95% CI of *R* = 0.50−0.99) and gram-negative soil bacteria (*n* = 5, 95% CI of *R* = 0.58−0.99). Within the gram-positive bacteria, actinomycete abundance did not change following disturbances (*n* = 14, 95% CI of *R* = 0.73−1.09; data not shown).

We found no evidence for publication bias among bacterial studies (Table A2). Similar to fungi, we could not use correlation or regression methods to test for publication bias in bacterial studies following biotic disturbance because there were only two observations.

### Recovery of microbial biomass following disturbances

**Figure 2:**
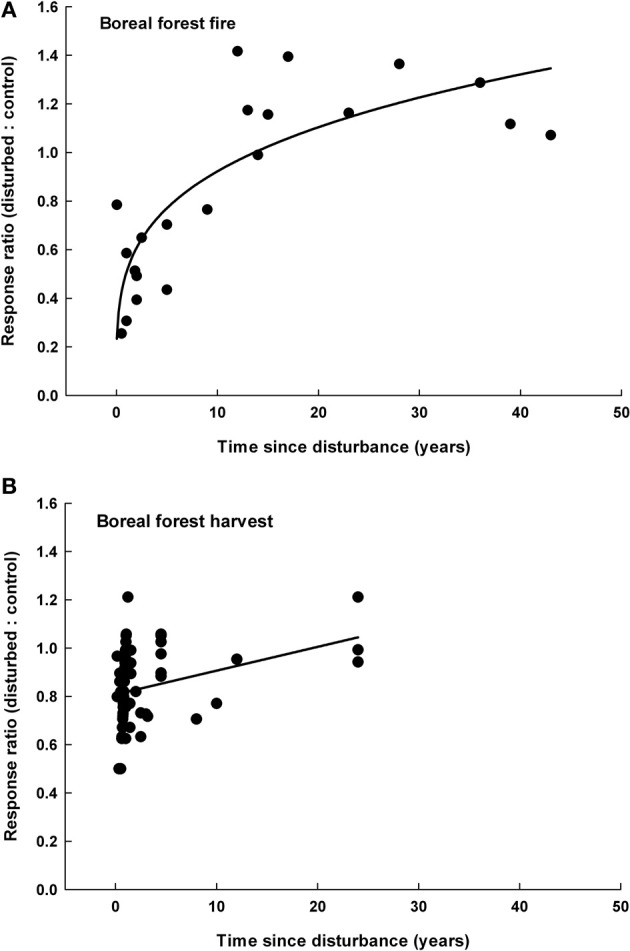
**The response ratio of microbial biomass as a function of the time since disturbance following boreal forest fires (A) and boreal forest harvesting (B)**. Response ratios significantly increased with time after boreal forest fires [*R* = 0.51 × (time since disturbance) ∧ 0.26, *n* = 21, *r*^2^ = 0.793, *P* < 0.0001] and boreal forest harvest (*R* = 0.01 × time since disturbance + 0.81, *n* = 32, *r*^2^ = 0.201, *P* = 0.010).

There was a significant positive relationship between the time since disturbance and the microbial biomass *R* following boreal forest fires (Figure 2A) and boreal forest harvesting (Figure 2B). Response ratios significantly increased as the time since fire increased in boreal forests (*n* = 21, *r*^2^ = 0.793, *P* < 0.0001). Similarly, microbial response ratios increased with the time since harvest in boreal forests (*n* = 32, *r*^2^ = 0.201, *P* = 0.010), and the relationship was linear.

We did not detect a significant relationship between microbial biomass response ratios and the time since disturbance for any other disturbance type and biome (data not shown). In addition, fungal and bacteria response ratios were not significantly related to the time since disturbance for any disturbance type and biome (data not shown).

### Basal respiration

**Figure 3:**
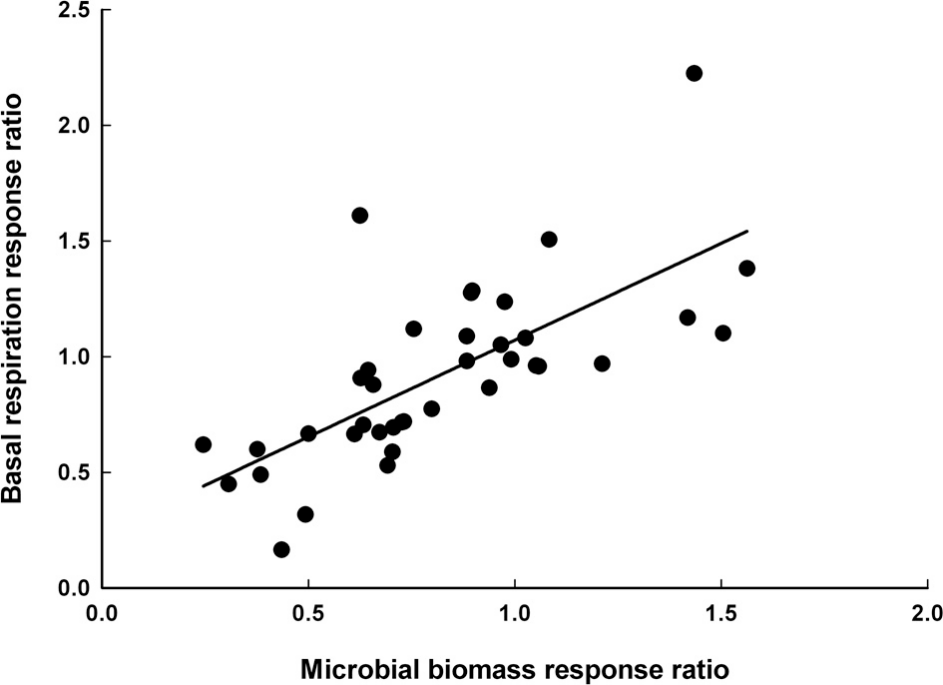
**The relationship between the response ratio of soil basal respiration and the response ratio of microbial biomass**. Each symbol designates one study. Line is the best-fit regression (basal respiration *R* = 0.84 × microbial biomass *R* + 0.24, *n* = 38, *r*^2^ = 0.492, *P* < 0.0001). The response of soil basal respiration is significantly related to the response of microbial biomass following disturbances.

A subset of the studies included in this meta-analysis reported changes in soil basal respiration following disturbance in addition to changes in microbial biomass measurements (*n* = 38). Across all studies that reported both, there was a significant positive correlation between the *R* of soil basal respiration and the *R* of microbial biomass (*r* = 0.702, *P* < 0.0001, Figure 3).

## Discussion

In this study, we conducted a meta-analysis of changes in soil microbial biomass in response to forest disturbances. We initially hypothesized that forest disturbances would reduce soil microbial biomass. In support of this hypothesis, we found that microbial biomass declined by an average of 29.4% after disturbance events (Table 1). The responses of soil fungi and bacteria to disturbance largely mirrored the response of the microbial community as a whole, and provide further support for the hypothesis that forest disturbances reduce soil microbial abundance. Although bacterial and fungal responses were less frequently studied than the response of the microbial community as a whole, these data imply that soil bacteria and fungi are affected by forest disturbances in a similar manner. Our data do not suggest that soil fungi are more sensitive to disturbance events than bacteria. We further hypothesized that abiotic disturbances would lead to greater reductions in microbial biomass than biotic disturbances. In support of this hypothesis, soil microbial responses significantly differed between abiotic and biotic disturbances. Fires, harvesting, and storms caused significant reductions in soil microbial biomass, while changes in microbial biomass following insect infestation and pathogen-induced tree mortality were non-significant (Figure 1A). Furthermore, bacterial and fungal abundances significantly increased following insect infestation (Figures 1B,C).

We propose two possible explanations for the differential effect of abiotic and biotic disturbances on soil microbial communities. First, abiotic disturbances typically involve higher levels of soil disruption during the disturbance event than biotic disturbances. For example, harvesting practices involve the use of logging equipment that can result in heavy soil compaction. Soil compaction alters soil pore space, potentially leading to impaired gas exchange, decreased soil drainage, and inhibition of soil microbial growth (Kabzems and Haeussler, 2005; Mariani et al., 2006). Forest fires cause soil disruption in the form of soil combustion and heating of the soil surface. Soil surface temperatures during forest fires can reach up to 600°C (Busse et al., 2005), which is well above the upper thermal limit of most microbial taxa (Debano et al., 1998). Storms cause soil disruption by uprooting trees, which can cause soil mixing and changes in soil microtopography (Ruel, 1995). These direct effects of abiotic disturbances on soil properties may in part explain the observed post-disturbance reductions in microbial biomass. In contrast, biotic disturbances do not typically involve immediate soil physical changes and are likely to have mostly indirect effects on soil properties (Hicke et al., 2012). Lower levels of soil physical disruption during biotic disturbances may in part explain the non-significant effect of these disturbances on soil microbial biomass.

In addition, abiotic and biotic disturbances differ in the amount and type of organic C remaining in ecosystems following the disturbance event and this may have consequences for soil microbial communities. Fires remove large amounts of organic C from ecosystems via the combustion of aboveground vegetation and soil organic matter (Amiro et al., 2001; Van Der Werf et al., 2010). The more labile components of soil organic matter may be preferentially volatized during fires (González-Pérez et al., 2004; Neff et al., 2005), leaving behind organic C that is more difficult for microbes to decompose. Harvesting also removes large amounts of organic C from forests, but can deposit fine woody debris on the soil surface. On the other hand, biotic disturbances are associated with smaller amounts of organic C removal from forests. Although insect or pathogen outbreaks may kill trees, they can also result in an influx of dead plant litter, insect feces, and dead insect biomass to forest soils (Lovett et al., 2002; Yang, 2004; Hicke et al., 2012). Higher amounts of organic C removal from forests during abiotic disturbances may cause C limitation of soil microbial growth, and thus reductions in soil microbial biomass. With our meta-analysis approach, we were unable to evaluate whether differences in soil physical disruption, organic C removal, or a combination of both factors, were responsible for the differential effect of abiotic and biotic disturbances on soil microbial communities. Future studies that are mechanistic rather than observational will make it possible to disentangle the factors that govern microbial responses to disturbance events.

While the mechanisms described above may explain the contrasting effects of abiotic and biotic disturbances that we observed, it is also important to consider that we found evidence for publication bias in studies of microbial biomass following biotic disturbances and in all fungal studies. The presence of publication bias suggests that the effects of disturbance that are reported in the literature may not be representative of all microbial responses. Moreover, microbial (*n* = 8), fungal (*n* = 2), and bacterial (*n* = 2) biomass responses to biotic disturbances were poorly represented in the literature. Therefore, the differences that we observed between abiotic and biotic disturbances may also be attributable to the scarcity of data on biotic disturbances.

In some cases, contrasts between disturbance agents revealed interesting differences in soil microbial responses. For example, clear cutting consistently reduced microbial abundance, but partial forest harvesting did not result in significant changes in total microbial biomass, fungal abundance, or bacterial abundance (Table 1). In comparison to clear cutting, partial harvesting is associated with lower levels of soil compaction and vegetation removal (Barg and Edmonds, 1999). Together these factors may explain the reduced impact of partial harvesting on belowground communities (Lindo and Visser, 2003). In addition, we found that gypsy moths and pine beetles had contrasting effects on soil microbial communities (Table 1). Pine beetle infestation reduced microbial biomass (95% CI of *R* = 0.367−0.646), while microbial biomass increased following gypsy moth defoliation (95% CI of *R* = 1.419−1.505). The differential effect of these insects on soil microbial biomass may be explained by their ecology. Gypsy moths are leaf-feeders that defoliate trees and reduce tree growth. However, gypsy moth feeding does not always kill trees. In contrast, pine beetles do not consume tree needles, but instead feed within the phloem and typically result in tree death (Hicke et al., 2012). Although represented by a limited number of studies, our results suggest that tree defoliating and tree killing insects may have contrasting effects of soil microbial communities and potentially forest C dynamics.

In addition to changing microbial biomass, disturbances may also alter the composition of soil microbial communities. Denaturing gradient gel electrophoresis and phospholipid fatty acid profiles have been used to detect broad changes in microbial community structure following harvesting and forest fires (Siira-Pietikainen et al., 2001; Waldrop and Harden, 2008). Next generation sequencing of environmental samples has made it possible to examine compositional changes in microbial communities following disturbances in greater detail. For example, Hartmann et al. (2012) found that harvesting significantly altered the composition of soil bacterial and fungal communities, with ectomycorrhizal taxa and actinobacteria being most sensitive to harvesting disturbance. Ectomycorrhizal fungi were also sensitive to forest fires in boreal forests, while ascomycete fungi increased in abundance following fire (Holden et al., 2013). These changes in microbial community structure following disturbance suggest that microbial species are differentially affected by disturbance. The functional consequences of compositional changes in soil microbial communities in response to disturbances require further testing. For instance, if plant symbiotic microbes are sensitive to disturbance, the ability of plants to re-establish following disturbances may be hindered. Changes in the composition of soil microbial communities following biotic disturbances have rarely been studied, but would greatly contribute to our knowledge of soil microbial responses to disturbances.

We found a significant positive relationship between the time since disturbance and microbial biomass responses following fire and harvesting in boreal forests (Figure 2). These results are consistent with our third hypothesis that post-disturbance changes in microbial biomass would weaken over time. Following both harvesting and fires in boreal forests, microbial responses were typically negative for the first 15 years following disturbance. This finding suggests that forest disturbances can have long-term consequences for belowground communities. Eddy covariance studies and ground-based vegetation surveys have found that primary productivity requires up to 10 years to recover following harvest and fires in boreal forests (Mack et al., 2008; Amiro et al., 2010; Goulden et al., 2011). In addition, post-fire reductions in soil C and soil organic matter can persist for at least 10 years following boreal forest fires (Johnson and Curtis, 2001; Treseder et al., 2004). Thus, the recovery of soil microbial biomass following harvesting and forest fires may be controlled by the recovery of forest primary productivity and soil organic matter accumulation. We found no evidence for a significant relationship between the time since disturbance and microbial abundance responses for any other disturbance type or biome. Although, the majority of the studies used in this meta-analysis assessed microbial responses to disturbance within 1 year of the disturbance event (Table A1). The paucity of long-term data may have limited our ability to detect significant relationships between microbial biomass responses and the time since disturbance. Additional long-term studies, especially following insect outbreaks and pathogen infection, are necessary to evaluate the belowground consequences of forest disturbances.

Classic ecosystem theory posits that soil microbial respiration increases following disturbance (Chapin et al., 2002; Harmon et al., 2011). Microbial respiration has long been assumed to increase following forest disturbance events because soil temperatures usually increase after disturbances and because disturbances can result in the deposition of plant litter and/or woody debris on the soil surface. Instead, we hypothesized that post-disturbance changes in microbial biomass would be associated with concurrent changes in microbial respiration. In support of our hypothesis, we found a significant positive correlation between the response of microbial biomass to disturbance and the response of soil basal respiration (Figure 3). Therefore, decreases in soil microbial biomass following abiotic disturbances may be accompanied by reductions in microbial respiration. This finding is in agreement with ecosystem-level studies that have measured microbial respiration following disturbance events and found post-disturbance decreases in microbial respiration (Amiro et al., 2003; Czimczik et al., 2006). Although, the microbial respiration data reported here were measured in the laboratory under standardized conditions. It is therefore possible that differences in soil conditions between disturbed and undisturbed forests may cause differences in microbial respiration in the field. However, any post-disturbance increases in microbial respiration would likely result from increases in mass-specific rates of respiration, since microbial abundance declined by an average of 29.4% following disturbances. Our understanding of changes in microbial respiration following disturbance would benefit from additional studies that combine *in situ* measurements of microbial respiration with detailed microbial community analyses.

In summary, we found that forests disturbances significantly reduced soil microbial biomass, but that responses differed by disturbance type. Microbial biomass responses were consistently negative following abiotic disturbances, but our data suggest that forest disturbances caused by biotic agents may have a neutral or positive effect on microbial abundance in soil. This contrast is potentially attributable to differences in soil physical disruption and organic C removal from forests between abiotic and biotic disturbances. Evidence for publication bias in biotic studies, and the overall paucity of data on soil microbial responses to biotic disturbances, may have also contributed to the patterns we observed. Further studies following biotic disturbances will help clarify their impact on soil microbial communities. We found that changes in soil microbial biomass following disturbances were significantly related to changes in microbial respiration. Disturbances are common in forest ecosystems and one indirect impact of climate warming in terrestrial ecosystems may be an increase in the frequency and severity of disturbance events in forests. Our results imply that these disturbance events can alter soil microbial biomass in forests, with corresponding consequences for microbial respiration and ecosystem C balance.

### Conflict of interest statement

The authors declare that the research was conducted in the absence of any commercial or financial relationships that could be construed as a potential conflict of interest.

## Appendix

**Table A1:** **A list of the studies used in meta-analyses**.

**Study**	**Disturbance type**	**Disturbance agent**	**Biome**	**Time since disturbance (*Y*)**	**Biomass method**	***R***	***lnR***
**MICROBES**							
Bååth et al., 1995	Fire	PF	BF	2.50	PLFA	0.65	−0.43
Bárcenas-Moreno et al., 2011	Fire	WF	TF	2.67	CF	0.38	−0.96
D'Ascoli et al., 2005	Fire	PF	WS	0.02	SIR	1.29	0.26
Dannenmann et al., 2011	Fire	WF	WS	0.50	CF	0.75	−0.29
Dangi et al., 2010	Fire	PF	WS	3.00	PLFA	0.69	−0.38
De Marco et al., 2005	Fire	PF	WS	40	CF	1.43	0.26
Dumontet et al., 1996	Fire	WF	TF	0.08	CF	0.75	−0.29
Fioretto et al., 2005	Fire	PF	WS	0.02	ATP	0.25	−1.39
Fenn et al., 1993	Fire	WF	WS	0.01	SIR	1.06	0.06
Fonturbel et al., 2012	Fire	PF	WS	0.01	SF	0.66	−0.42
Fritze et al., 1993	Fire	PF	BF	0.01	CF	0.78	−0.24
Fritze et al., 1994	Fire	PF	BF	2.00	CF	0.39	−0.93
Gömöryová et al., 2008	Fire	PF	WS	0.01	CF	1.08	0.08
Goberna et al., 2012	Fire	WF	TF	0.96	Micro	0.59	−0.52
Grady and Hart, 2006	Fire	WF	TF	7.00	CF	0.38	−0.98
Hamman et al., 2007	Fire	WF	TF	1.00	PFLA	0.84	−0.18
Kara and Bolat, 2009	Fire	WF	TF	0.17	CF	0.98	−0.02
Leduc and Rothstein, 2007	Fire	WF	TF	4.50	CF	0.61	−0.49
Litton et al., 2003	Fire	WF	TF	13.00	CF	0.44	−0.83
Mabuhay et al., 2006	Fire	WF	TF	0.01	CF	0.04	−3.12
Palese et al., 2004	Fire	PF	WS	1.00	CF	0.37	−1.00
Pietikäinen and Fritze, 1995	Fire	PF	BF	1.00	CF	0.31	−1.18
Prieto-Fernández et al., 1998	Fire	WF	TF	0.01	CF	0.04	−3.14
Rutigliano et al., 2007	Fire	PF	WS	0.02	CF	1.50	0.41
Smith et al., 2008	Fire	WF	BF	0.50	CF	0.25	−1.37
Swallow et al., 2009	Fire	PF	BF	1.83	CF	0.51	−0.67
Waldrop and Harden, 2008	Fire	WF	BF	5.00	CF	0.43	−0.83
Arunachalam et al., 1996	Harvest	CC	TF	1.08	CF	0.19	−1.66
Bååth et al., 1995	Harvest	CC	BF	3.17	PLFA	0.72	−0.33
Barbhuiya et al., 2004	Harvest	CC	TrF	7.00	CF	0.37	−1.00
Barbhuiya et al., 2004	Harvest	PH	TrF	8.00	CF	0.58	−0.54
Barg and Edmonds, 1999	Harvest	CC	TF	3.50	CF	1.07	0.06
Barg and Edmonds, 1999	Harvest	PH	TF	3.50	CF	1.13	0.13
Bradley et al., 2001	Harvest	CC	TF	4.00	SIR	0.67	−0.40
Bradley et al., 2001	Harvest	PH	TF	4.00	SIR	0.70	−0.35
Busse et al., 2006	Harvest	CC	TF	6.00	SIR	0.47	−0.76
Chang et al., 1995	Harvest	CC	TF	3.00	CF	0.63	−0.46
Chatterjee et al., 2008	Harvest	CC	TF	15.00	PLFA	0.83	−0.19
Edmonds et al., 2000	Harvest	CC	TF	3.50	CF	1.19	0.18
Entry et al., 1986	Harvest	CC	TF	2.00	CF	1.02	0.02
Forge and Simard, 2000	Harvest	CC	TF	2.00	CF	0.51	−0.67
Grady and Hart, 2006	Harvest	PH	TF	8.00	CF	0.64	−0.44
Hannam et al., 2006	Harvest	CC	BF	4.50	PLFA	0.88	−0.12
Hannam et al., 2006	Harvest	PH	BF	4.50	PLFA	0.89	−0.11
Hassett and Zak, 2005	Harvest	CC	BF	10.00	PLFA	0.77	−0.26
Hazlett et al., 2007	Harvest	CC	BF	2.00	CF	0.82	−0.20
Holmes and Zak, 1999	Harvest	CC	BF	1.00	CF	1.31	0.27
Houston et al., 1998	Harvest	CC	BF	8.00	SIR	0.71	−0.35
Lapointe et al., 2005	Harvest	CC	BF	1.50	SIR	0.94	−0.06
Leduc and Rothstein, 2007	Harvest	CC	TF	4.50	CF	0.69	−0.37
Lindo and Visser, 2003	Harvest	CC	BF	2.50	SIR	0.73	−0.31
Maassen et al., 2006	Harvest	PH	TF	5.00	SIR	1.56	0.45
Moore-Kucera and Dick, 2008	Harvest	CC	TF	8.00	PLFA	0.66	−0.42
Pérez-Batallón et al., 2001	Harvest	CC	TF	1.00	CF	0.99	−0.01
Pietikäinen and Fritze, 1995	Harvest	CC	BF	3.00	CF	0.73	−0.32
Saynes et al., 2012	Harvest	PH	TrF	1.00	CF	0.63	−0.47
Siira-Pietikäinen et al., 2001	Harvest	CC	BF	0.17	SIR	0.97	−0.03
Siira-Pietikäinen et al., 2001	Harvest	PH	BF	0.17	SIR	0.80	−0.22
Smith et al., 2008	Harvest	CC	BF	0.50	CF	0.82	−0.20
Tan et al., 2008	Harvest	PH	BF	24	CF	1.21	0.19
Taylor et al., 1999	Harvest	CC	TF	3.21	Count	0.88	−0.13
Wright and Coleman, 2002	Harvest	CC	TF	0.25	CF	0.97	−0.03
Zhao et al., 2011	Harvest	CC	TrF	0.33	PLFA	1.12	0.11
Zu et al., 2009	Harvest	CC	TF	8.00	CF	1.10	0.09
Gömöryová et al., 2008	Storm	WT	TF	0.96	Micro	0.54	−0.61
Tsai et al., 2007	Storm	TY	TrF	0.01	CF	0.24	−1.41
Wright and Coleman, 2002	Storm	HU	TF	0.25	CF	1.04	0.04
Bogorodskaya et al., 2009	Insect	GM	BF	0.13	SIR	1.41	0.35
Le Mellec and Michalzik, 2008	Insect	PL	TF	0.08	CF	1.03	0.03
Xiong et al., 2011	Insect	PB	TF	2.00	CF	0.60	−0.52
Xiong et al., 2011	Insect	PB	TF	4.00	CF	0.67	−0.41
Cromack et al., 1991	Pathogen	PW	TF	2.00	CF	0.54	−0.61
Mabuhay and Nakagoshi, 2012	Pathogen	PWD	TF	2.00	CF	1.55	0.44
**FUNGI**							
Bååth et al., 1995	Fire	PF	BF	2.50	PLFA	0.37	−0.99
Bárcenas-Moreno et al., 2011	Fire	WF	TF	2.67	PLFA	0.33	−1.10
Capogna et al., 2009	Fire	PF	WS	0.23	Count	0.42	−0.87
D'Ascoli et al., 2005	Fire	PF	WS	0.02	Microsc	0.60	−0.51
Dangi et al., 2010	Fire	PF	WS	3.00	PLFA	0.34	−1.08
Esquilín et al., 2007	Fire	SB	TF	0.02	Microsc	0.89	−0.12
Fritze et al., 1994	Fire	PF	BF	2.00	Ergosterol	0.42	−0.87
Hamman et al., 2007	Fire	WF	TF	1.00	PLFA	0.53	−0.64
Kara and Bolat, 2009	Fire	WF	TF	0.17	Count	0.62	−0.47
Mabuhay et al., 2006	Fire	WF	TF	0.01	Count	0.03	−3.47
Pietikäinen and Fritze, 1995	Fire	PF	BF	1.00	Ergosterol	0.30	−1.21
Rutigliano et al., 2007	Fire	PF	WS	0.02	Microsc	0.61	−0.50
Waldrop and Harden, 2008	Fire	WF	BF	5.00	qPCR	0.40	−0.93
Bååth et al., 1995	Harvest	CC	BF	3.17	PLFA	0.41	−0.89
Barbhuiya et al., 2004	Harvest	CC	TrF	7.00	Count	0.45	−0.79
Barbhuiya et al., 2004	Harvest	PH	TrF	8.00	Count	0.45	−0.79
Carter et al., 2002	Harvest	CC	TF	0.50	Count	1.00	0.00
Chatterjee et al., 2008	Harvest	CC	TF	15.00	PLFA	0.47	−0.76
Forge and Simard, 2000	Harvest	CC	TF	2.00	Microsc	0.47	−0.76
Hannam et al., 2006	Harvest	CC	BF	4.50	PLFA	0.88	−0.13
Hannam et al., 2006	Harvest	PH	BF	4.50	PLFA	1.00	0.00
Hassett and Zak, 2005	Harvest	CC	BF	10.00	PLFA	0.85	−0.16
Hernesmaa et al., 2008	Harvest	CC	BF	0.75	Count	1.02	0.02
Maassen et al., 2006	Harvest	PH	TF	5.00	PLFA	1.6	0.47
Moore-Kucera and Dick, 2008	Harvest	CC	TF	8.00	PLFA	0.49	−0.70
Pietikäinen and Fritze, 1995	Harvest	CC	BF	3.00	Ergosterol	0.68	−0.39
Stadler et al., 2006	Insect	HWA	TF	0.08	Count	1.19	0.17
**BACTERIA**							
Bååth et al., 1995	Fire	PF	BF	2.50	PLFA	0.73	−0.31
Bárcenas-Moreno et al., 2011	Fire	WF	TF	2.67	PLFA	0.43	−0.85
Esquilín et al., 2007	Fire	SB	TF	0.02	Microsc	0.77	−0.26
Hamman et al., 2007	Fire	WF	TF	1.00	PLFA	0.94	−0.06
Kara and Bolat, 2009	Fire	WF	TF	0.17	Count	5.73	1.75
Bååth et al., 1995	Harvest	CC	BF	3.17	PLFA	0.76	−0.28
Barbhuiya et al., 2004	Harvest	CC	TrF	7.00	Count	0.57	−0.57
Barbhuiya et al., 2004	Harvest	PH	TrF	8.00	Count	0.63	−0.46
Carter et al., 2002	Harvest	CC	TF	0.50	Count	1.00	0.00
Chatterjee et al., 2008	Harvest	CC	TF	15.00	PLFA	0.84	−0.17
Forge and Simard, 2000	Harvest	CC	TF	2.00	Microsc	0.98	−0.02
Maassen et al., 2006	Harvest	PH	TF	5.00	PLFA	1.52	0.42
Moore-Kucera and Dick, 2008	Harvest	CC	TF	8.00	PLFA	0.66	−0.42
Stadler et al., 2006	Insect	HWA	TF	0.08	Count	1.10	0.10
**GRAM-NEGATIVE BACTERIA**							
Dangi et al., 2010	Fire	PF	WS	3.00	PLFA	0.69	−0.37
Chatterjee et al., 2008	Harvest	CC	TF	15.00	PLFA	0.96	−0.04
Hassett and Zak, 2005	Harvest	CC	BF	10.00	PLFA	1.01	0.01
Moore-Kucera and Dick, 2008	Harvest	CC	TF	8.00	PLFA	0.99	0.00
Mabuhay and Nakagoshi, 2012	Pathogen	PWD	TF	2.00	Count	0.46	−0.77
**GRAM-POSITIVE BACTERIA**							
Dangi et al., 2010	Fire	PF	WS	3.00	PLFA	0.86	−0.15
Chatterjee et al., 2008	Harvest	CC	TF	15.00	PLFA	0.62	−0.47
Hassett and Zak, 2005	Harvest	CC	BF	10.00	PLFA	1.00	0.00
Moore-Kucera and Dick, 2008	Harvest	CC	TF	8.00	PLFA	1.10	0.10
Mabuhay and Nakagoshi, 2012	Pathogen	PWD	TF	2.00	Count	0.35	−1.04
**ACTINOMYCETES**							
Bárcenas-Moreno et al., 2011	Fire	WF	TF	2.67	PLFA	2.84	1.04
Dangi et al., 2010	Fire	PF	WS	3.00	PLFA	0.66	−0.42
Carter et al., 2002	Harvest	CC	TF	0.50	Count	1.00	0.00
Chatterjee et al., 2008	Harvest	CC	TF	15.00	PLFA	0.88	−0.13
Hannam et al., 2006	Harvest	CC	BF	4.50	PLFA	1.06	0.06
Hannam et al., 2006	Harvest	PH	BF	4.50	PLFA	1.00	0.00
Hassett and Zak, 2005	Harvest	CC	BF	10.00	PLFA	0.98	−0.03
Maassen et al., 2006	Harvest	PH	TF	5.00	PLFA	1.17	0.15
Moore-Kucera and Dick, 2008	Harvest	CC	TF	8.00	PLFA	1.11	0.11
Mabuhay and Nakagoshi, 2012	Pathogen	PWD	TF	2.00	Count	0.29	−1.23

*PF, prescribed fire; SB, slash burn; WF, wildfire; CC, clear cut; PH, partial harvest; HU, hurricane; WT, wind throw; TY, typhoon; GM, gypsy moth; HWA, hemlock wooly adelgid; PB, pine beetle; PL, pine lappet; PW, Phellinus weirii infection; PWD, pine wilt disease; BF, boreal forest; TF, temperate forest; TrF, tropical forest; WS, woodland/shrubland, CF, chloroform fumigation; Count, dilution plate count; Micro, microwave irradiation; Microsc, microscopy; PLFA, phospholipid fatty acid; qPCR, quantitative PCR; SIR, substrate-induced respiration*.

**Table A2:** **Outcomes of test for publication bias**.

**Organism**	**Group**	**Kendall's tau rank correlation**	**Spearman rank correlation**	**Egger's regression**
Microbes	All microbe studies	τ: −0.084	ρ: −0.099	Intercept: −5.62
		*P*: 0.249	*P*: 0.360	*P*: 0.136
	All abiotic	τ: −0.038	ρ: −0.037	Intercept: −5.99
		*P*: 0.618	*P*: 0.746	*P*: 0.124
	All abiotic	τ: −0.038	ρ: −0.037	Intercept: −5.99
		*P*: 0.618	*P*: 0.746	*P*: 0.124
	All biotic	τ: −0.512	ρ: −0.655	Intercept: −5.71
		*P*: 0.076	*P*: 0.078	***P*: 0.029**
Fungi	All fungi studies	τ: −0.314	ρ: −0.416	Intercept: −7.23
		***P*: 0.008**	***P*: 0.013**	*P*: 0.377
	All abiotic	τ: −0.425	ρ: −0.560	Intercept: −5.61
		***P*: 0.001**	***P*: 0.001**	*P*: 0.230
	All biotic	n.a.	n.a	n.a.
Bacteria	All bacteria studies	τ: 0.033	ρ: 0.062	Intercept: −2.67
		*P*: 0.855	*P*: 0.812	*P*: 0.537
	All abiotic	τ: 0.082	ρ: 0.144	Intercept: −4.00
		*P*: 0.669	*P*: 0.608	*P*: 0.446
	All biotic	n.a.	n.a.	n.a.

*Tests could not be performed on biotic studies within fungi and bacteria because not enough studies were present. Boldface type indicates significance at P < 0.05*.
